# Epidemiology of Mortality Related to Chagas' Disease in Brazil, 1999–2007

**DOI:** 10.1371/journal.pntd.0001508

**Published:** 2012-02-14

**Authors:** Francisco Rogerlândio Martins-Melo, Carlos Henrique Alencar, Alberto Novaes Ramos, Jorg Heukelbach

**Affiliations:** 1 Department of Community Health, School of Medicine, Federal University of Ceará, Fortaleza, Brazil; 2 School of Public Health, Tropical Medicine and Rehabilitation Sciences, Anton Breinl Centre for Public Health and Tropical Medicine, James Cook University, Townsville, Australia; National Institutes of Health, United States of America

## Abstract

**Background:**

Chagas' disease is an important neglected public health problem in many Latin American countries, but population-based epidemiological data are scarce. Here we present a nationwide analysis on Chagas-associated mortality, and risk factors for death from this disease.

**Methodology/Principal Findings:**

We analyzed all death certificates of individuals who died between 1999 and 2007 in Brazil, based on the nationwide Mortality Information System (a total of 243 data sets with about 9 million entries). Chagas' disease was mentioned in 53,930 (0.6%) of death certificates, with 44,537 (82.6%) as an underlying cause and 9,387 (17.4%) as an associated cause of death. Acute Chagas' disease was responsible for 2.8% of deaths. The mean standardized mortality rate was 3.36/100.000 inhabitants/year. Nationwide standardized mortality rates reduced gradually, from 3.78 (1999) to 2.78 (2007) deaths/year per 100,000 inhabitants (−26.4%). Standardized mortality rates were highest in the Central-West region, ranging from 15.23 in 1999 to 9.46 in 2007 (−37.9%), with a significant negative linear trend (p = 0.001; R^2^ = 82%). Proportional mortality considering multiple causes of death was 0.60%. The Central-West showed highest proportional mortality among regions (2.17%), with a significant linear negative trend, from 2.28% to 1.90% (−19.5%; p = 0.001; R^2^ = 84%). There was a significant increase in the Northeast of 38.5% (p = 0.006; R^2^ = 82%). Bivariable analysis on risk factors for death from Chagas' disease showed highest relative risks (RR) in older age groups (RR: 10.03; 95% CI: 9.40–10.70; p<0.001) and those residing in the Central-West region (RR: 15.01; 95% CI: 3.90–16.22; p<0.001). In logistic regression analysis, age ≥30 years (adjusted OR: 10.81; 95% CI: 10.03–10.65; p<0.001) and residence in one of the three high risk states Minas Gerais, Goiás or the Federal District (adjusted OR: 5.12; 95% CI: 5.03–5.22, p<0.001) maintained important independent risk factors for death by Chagas' disease.

**Conclusions/Significance:**

This is the first nationwide population-based study on Chagas mortality in Brazil, considering multiple causes of death. Despite the decline of mortality associated with Chagas' disease in Brazil, the disease remains a serious public health problem with marked regional differences.

## Author Summary

American trypanosomiasis (Chagas' disease) is a parasitic disease which remains a public health problem in Latin America, but studies investigating the dynamics in populations under risk are scarce. We conducted a nation-wide study based on about 9 million Brazilian death certificates from 1999–2007. Epidemiological characteristics of Chagas-related deaths, temporal trends and associated factors were investigated. Chagas' disease was mentioned in about 54,000 (0.6%) death certificates, resulting in a mean standardized mortality rate of 3.36/100,000 inhabitants/year. Nationwide mortality rates reduced gradually, from 3.78 (1999) to 2.78 (2007) deaths/year/100,000 inhabitants (−26.4%). The vast majority of deaths (97.2%) were caused by chronic form of the disease. There were marked regional differences: mortality rates were highest in the Central-West region. In contrast to the rest of the country, there was a significant increase in the Northeast (38.5%). Risk factors independently associated with Chagas mortality were age >30 years (adjusted OR = 10.60; 9.90–11.33, p<0.001) and residing in the states of Minas Gerais, Goiás or Federal District (adjusted OR = 4.89, 4.81–4.98, p<0.001). We performed the first nationwide population-based study on Chagas mortality, considering multiple causes of death in Brazil. A comprehensive overview of mortality associated with Chagas' disease is provided. Chagas' disease is still a major public health problem in the country.

## Introduction

American trypanosomiasis (Chagas' disease) is an anthropozoonic vector-borne parasitic infection caused by the protozoan parasite *Trypanosoma cruzi* [1]–[4]. Other important infection routes include blood transfusion, vertical transmission, organ transplantation, and oral transmission [1], [2]. There are acute (often asymptomatic) and chronic phases of the disease. In the case of untreated acute disease, the infection may persist for many years and even decades. Chronic disease manifests as indeterminate, cardiac, gastrointestinal and neural forms [2]. As a chronic condition, the infection may be associated with other chronic diseases further increasing mortality [5].

Chagas' disease is the sixth most important tropical infection in the world in terms of global burden of disease [6], with high social and economic impact throughout the endemic area [7]–[9]. Chronic Chagas' disease is a public health problem faced by many Latin American countries, from Mexico in the north to Argentina in the south. There are about 15 to 18 million people infected in Latin America, and approximately 100 million are at potential risk for infection [3], [8]. An estimated 14,000 people die from the disease each year worldwide [3]. Single autochthonous cases were notified in the southern United States, but thousands are estimated [4], [10].

Chronic cardiac disease is the leading cause of disability-adjusted life years (DALYs) lost in young economically active adults in endemic countries, with considerable social, public health and economic consequences [2]. The migration of infected individuals to non-endemic countries has become an emerging public health problem in these regions, mainly in the United States and European countries [3]. The principal means of transmission in these countries are from blood transfusion, organ transplantation and mother-to-child transmission [11].

In Brazil, the number of new Chagas' disease cases has been reduced dramatically in recent years, owing mainly to reduction of vector transmission (mostly by the kissing bug *Triatoma infestans*) and control of infection by blood transfusion [8]–[11]. However, there is still a plethora of people living with chronic forms of the disease. About 2 to 3 million people are estimated to be infected in Brazil [7], [12], 600,000 of them with chronic heart or digestive complications, causing death in about 5,000 individuals each year [13].

Despite the importance for health policy and planning, mortality statistics from endemic countries have not yet been used systematically to assess the impact of control measures against Chagas' disease [14]. To fill this gap, we present the first Brazilian nationwide population-based study on Chagas mortality, using multiple causes of death, as available on death certificates. We analyzed mortality trends over time, geographical regions mostly affected, and factors associated with the occurrence of death caused by Chagas' disease.

## Methods

### Study area and data sources

Brazil has a population of approximately 190 million inhabitants. The country is divided into 26 States and one Federal District. The Federation is further grouped into five major regions (North, Northeast, Southeast, South and Central-West) with different geographic, economic and cultural characteristics.

We analyzed all death certificates of individuals who died between 1999 and 2007 in Brazil. As data source we used the Brazilian death certificates, standardized by the Mortality Information System (*Sistema de Informação de Mortalidade -* SIM) of the Ministry of Health, a national electronic database. SIM data are public domain and were obtained from the website of the Department of the Unified Health System, DATASUS (http://tabnet.datasus.gov.br/tabdata/sim/dados/cid10_indice.htm). Death certificates contain demographic (age, gender, education, race, marital status, municipality of residence and municipality of occurrence of death) and clinical information (underlying and associated causes of death). It is the physicians' responsibility to complete the death certificate forms. Until 1995, reference codes were based on the International Classification of Diseases (ICD) in its 9th revision, and after 1996 in its 10th revision [15].

We identified Chagas-related deaths using category B57 (“Chagas' disease”) including all subcategories (B57.0 to B57.5) which represent both acute and chronic clinical forms, according to the Tenth Revision of the International Statistical Classification of Diseases and Related Health Problems (ICD-10) [15].

Population estimates were obtained from the Brazilian Institute of Geography and Statistics (*Instituto Brasileiro de Geografia e Estatística* - IBGE) based on a national population census in 2000 and yearly official estimates (1999–2007). The census is a fundamental source of information from the entire Brazilian population, and data collection and analysis are subject to supervision and quality control.

This study was based on publicly available secondary anonymous data, with no possibility of identification of individuals. Thus, approvement of the study by a Ethical Review Board was not necessary.

### Data processing and analysis

A total 243 data sets with about 9 million entries were downloaded and processed (one data set for each of the 27 federal states and each of the 9 years, from 1999–2007). In a first step, we checked data sets for completeness in relation to the total number of deaths. Field codes from different data sets were standardized and variables not considered in the analysis eliminated. We then identified all death certificates in which Chagas' disease was recorded in any line of the certificate as cause of death (both underlying and associated causes). We created new variables for causes of death, as in many cases more than one cause was noted in a line.

Then, Chagas-related mortality rates for multiple causes of death and underlying causes of death from 1999–2007 were calculated. These mortality rates were calculated by dividing the number of deaths in each calendar year by the population, and presented per 100,000. We calculated the standardization of mortality rates by age using the direct method, considering the Brazilian population in 2003 as standard [16]. The proportional mortality rate was calculated by dividing the number of deaths from Chagas' disease by the total number of deaths multiplied by 100. We present standardized specific mortality and proportional mortality rates, stratified by region of residence, year of occurrence of death, and age group. Mortality analysis in this study focuses on multiple causes of death (including both underlying and any other causes), and on the usual approach of underlying causes of death (the disease or condition, which led directly to the death) [16].

Analysis of time trends of mortality rates was performed using polynomial regression models. The polynomial model aimed to find the curve that would best fit the data in order to describe the relationship between the dependent variable (mortality associated with Chagas' disease) and the independent variable (year of death). We first made scatter diagrams of indicators of mortality and the years of death, to visualize the mathematical function that would best represent the relationship between variables. From this observation, we estimated the regression models. We tested the following regression models where the values of Y and X are the dependent and independent variables respectively and β_0_, β_1_, β_2_ and β_3_ are the coefficients of regression [15]: a) linear (1st order): Y = β_0_+β_1_X; b) 2nd order: Y = β_0_+β_1_X+β_2_X^2^; c) third order: Y = β_0_+β_1_X+β_2_X^2^+β_3_X^3^; d) exponential: Y = e^β0+β1X^. The choice of the best-fitting model was based on an analysis of the diagram, the coefficient of determination (R^2^), the statistical significance and residual analysis (true homoscedasticity assumption). When the trend was not statistically significant, the model was chosen according to the coefficient of determination and when two models were similar, the statistical point of view, we chose the simplest model (lowest order). Trends were considered statistically significant when the models showed p<0.05.

We further performed bivariable and multivariable analysis to identify association between demographic variables available (gender, age, race, state and region of residence, residence in state capital, year of death) and death due to Chagas' disease. We calculated relative risks (RR) with their respective 95% confidence intervals and applied the chi-squared test to estimate significance of the differences between relative frequencies. In logistic regression analysis the variables included were gender, age ≥30 years, and residence in a high risk state Minas Gerais, Goiás or Federal District. The cut-off point of 30 years was based on the natural history of the disease and disease control measures implemented in the 1970s and 1980s [1], [9]–[13]. Region of residence and place of occurrence were excluded due to collinearity with the variable on high risk states included. The information on race/color lacked information in more than 10% of cases and was thus not included; in addition, information on skin color is not standardized in Brazil, and interpretation of this variable is limited.

Calculation of the indicators and the preparation of tables and figures were performed in Microsoft Excel spreadsheets. We used SPSS for Windows version 15.0 (Statistical Package for the Social Sciences; SPSS Corporation, Chicago, USA) for calculation of polynomial regression models. Bivariable and multivariable statistical analysis was performed using the programs STATA version 11 (Stata Corporation, College Station, USA) and Epi Info for Windows version 3.5.1 (Centers for Disease Control and Prevention, Atlanta, USA).

## Results

Of the 8,942,217 deaths occurring in Brazil between 1999 and 2007, acute or chronic Chagas' disease was mentioned in 53,930 (0.6%) cases in any part of the death certificates, with 44,543 (82.6%) as an underlying cause and 9,387 (17.4%) as an associated cause of death. The resulting mean standardized mortality rate related to Chagas' disease considering all fields of the death certificates was 3.36/100,000 inhabitants/years. This is 21% higher than the mortality rate considering merely underlying cause of death (2.78 deaths/100,000 inhabitants/year). The mean number of deaths related to Chagas' disease was 5,992 per year, ranging from 5,573 in 1999 to 6,227 in 2006.

Chronic forms (B57.2–B57.5) were identified in the vast majority of deaths (n = 43,302; 97.2%). Deaths related to acute Chagas' disease (B57.0 and B57.1) represented only 2.8% (n = 1,241), but increased by 24.4% in the study period. Death due to acute Chagas' disease was most common in the North (Amazon) region of the country (11%; n = 64/581).

Individuals who died from Chagas' disease were predominantly males (57%), aged >60 years (62.8%) and residents in the Southeast region (53.6%). The age at death ranged from 0 to 109 years with a median of 69 years (mean = 66.9 years, SD ± 14.6); 77.1% were living in other cities than state capitals, but only 32.3% died there. This means that a considerable number of Chagas' disease patients did not live in the capitals, but died there.

### Standardized specific mortality rates

**Figure 1:**
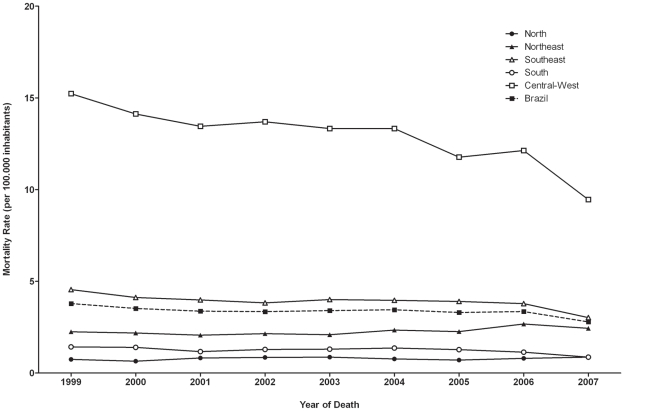
Standardized mortality rates related to Chagas' disease in Brazil and regions, 1999 to 2007.

Trends of standardized specific mortality rates, stratified by Brazilian regions, are shown in Figure 1. Nationwide standardized mortality rates related to Chagas' disease reduced gradually from 1999 to 2007 by 26.4%, from 3.78 to 2.78 deaths/100,000 inhabitants (Figure 1). Chagas as underlying cause of death decreased by 33.2%, from 3.28 to 2.19 deaths/100,000 inhabitants. On the other hand, there was a relative increase by 20.4% of the rate of associated causes of death, from 0.49 to 0.59 per 100,000 inhabitants between 1999 and 2007.

There were distinct regional patterns. The standardized mortality rates were highest in the Central-West region, with rates ranging from 15.23 in 1999 to 9.46 in 2007, a decrease of 37.9%. Polynomial regression shows that during the study period this decrease was linear with a significantly high R^2^ value of 82% (Table 1). Southeast and South regions showed a decrease of 28.8% and 41.8%, respectively (Figure 1). In contrast, mortality rates increased significantly during the study period in the Northeast region. The North presented the lowest coefficients, with a stabilization (Figure 1, Table 1).

**Table 1:** Trends of mortality indicators related to Chagas' disease, Brazil and regions, from 1999 to 2007.

Indicator	Model*	R^2^ **	p	Trend
**Standardized specific mortality rate**				
Brazil	Y = 3.37−0.076x	0.63	0.011	Descending
North	Y = 0.79+0.011x	0.15	0.309	Stabilization
Northeast	Y = 2.18+0.047x+0.013x**^2^**	0.64	0.047	Crescent
Southeast	Y = 3.90−0.119x	0.66	0.007	Descending
South	Y = 1.25−0.045x	0.52	0.028	Descending
Central-West	Y = 12.95−0.547x	0.82	0.001	Descending
**Proportional mortality rate**				
Brazil	Y = 0.61−0.003x	0.42	0.059	Stabilization
North	Y = 0.14+0.003x	0.28	0.146	Stabilization
Northeast	Y = 0.41+0.011x+0.004x**^2^**	0.82	0.006	Crescent
Southeast	Y = 0.67−0.006x	0.40	0.069	Stabilization
South	Y = 0.22−0,005x	0.42	0.058	Stabilization
Central-West	Y = 2.18−0,064x	0.84	0.001	Descending

*Model: y = standardized mortality rates (per 100 000 inhabitants) or proportional mortality related to Chagas' disease, considering multiple causes of death, x = year of death - year average of the period studied (2003).

**R^2^ = coefficient of determination.

### Proportional mortality rates

**Figure 2:**
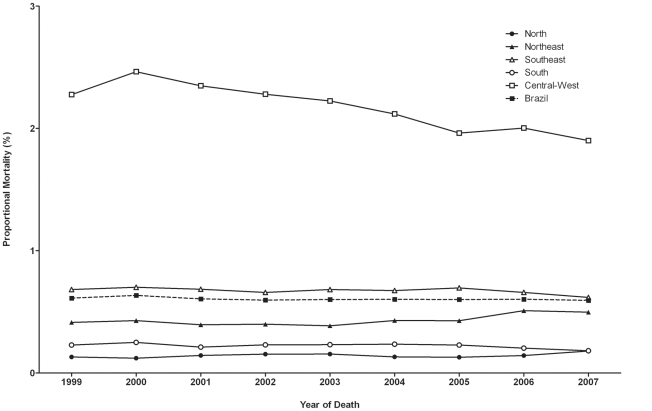
Proportional mortality related to Chagas' disease in Brazil and regions, from 1999 to 2007.

Proportional mortality rates over time, stratified by Brazilian regions, are shown in Figure 2. Similar to specific rates, the inclusion of multiple causes of death as compared to underlying causes increased mortality rate by 20% (0.50% vs. 0.60%). There was a progressive decrease of proportional mortality as the underlying cause in Brazil, from 0.53% to 0.45% (15% decline), but an increase of mortality as associated cause, from 0.08% to 0.14% (75% increase), resulting in an overall stabilization of proportional mortality considering multiple causes of death *i.e*. all mentions (Figure 2). The Central-West showed highest proportional mortality among regions (2.17%), with a significant linear decreasing trend of multiple causes, from 2.28% to 1.90% (19.5%, R^2^ = 84%) between 1999 and 2007 (Figure 2, Table 1). In contrast, there was a significant increase in the Northeast of 38.5% (R^2^ = 82%). The three other regions showed stabilization of proportional mortality (Table 1, Figure 2).

### Factors associated with mortality related to Chagas' disease

Bivariable analysis of variables associated with Chagas' disease as a multiple cause of death is presented in Table 2. All variables available from death certificates were significantly associated with Chagas mortality. Highest relative risks were found in older age groups and those residing in the Central-West. The result of logistic regression is depicted in Table 3. Similarly, age ≥30 years and residence in one of the three high risk states maintained important independent risk factors for death by Chagas' disease.

**Table 2:** Bivariable analysis of factors associated with death caused by Chagas' disease, Brazil 1999–2007.

Variables	Death from Chagas'disease		RR	95% confidence interval	P value
	n	%			
**Gender** *					
Female	23,172	0.622	Ref.		
Male	30,748	0.596	0.96	0.94–0.97	<0.001
**Age group (years)** *					
<15	118	0.016	Ref.		
15–29	815	0.128	7.59	6.25–9.20	<0.001
30–39	2,333	0.436	25.76	21.41–31.00	<0.001
40–49	5,783	0.746	43.93	36.61–52.71	<0.001
50–59	10,929	1.047	61.44	51.25–73.66	<0.001
60–69	13,686	0.980	57.60	48.05–69.04	<0.001
70 or more	20,214	0.536	31.67	26.43–37.95	<0.001
**Age <30 years** *					
Yes	933	0.069	Ref.		
No	52,945	0.704	10.03	9.40–10.70	<0.001
**Race/Color** *					
Caucasian	25,067	0.531	Ref.		
Indigenous	54	0.268	0.50	0.39–0.66	<0.001
Yellow	344	0.470	0.89	0.80–0.98	0.026
Black	5,341	0.879	1.65	1.60–1.69	<0.001
Brown	16,017	0.677	1.27	1.25–1.30	<0.001
**Region of residence**					
North	676	0.144	Ref.		
South	3,165	0.223	1.54	1.42–1.68	<0.001
Northeast	9,618	0.435	3.00	2.78–3.25	<0.001
Central-West	11,533	2.213	15.01	13.90–16.22	<0.001
Southeast	28,938	0.677	4.66	4.32–5.03	<0.001
**Federal Units**					
Other states	30,038	0.391	Ref.		
Minas Gerais, Goiás and Federal District	23,892	1.952	4.91	4.82–4.99	<0.001
**Residence in state capital**					
Yes	12,371	0.544	Ref.		
No	41,559	0.628	1.15	1.13–1.18	<0.001
**Occurrence in state capital**					
Yes	36,524	0.614	1.04	1.02–1.06	<0.001
No	17,406	0.591	Ref.		

*Data not available in all cases.

Total number of deaths: 8,942,217.

**Table 3:** Logistic regression analysis of variables independently associated with death caused by Chagas' disease, Brazil 1999–2007.

Variable	adjusted OR	95% confidence interval	P value
Age ≥30 years	10.60	9.90–11.33	<0.001
Living in Minas Gerais, Goiás or Federal District	4.89	4.81–4.98	<0.001
Living outside urban area of state capital	1.04	1.02–1.06	<0.001
Male gender	1.02	1.00–1.03	0.045

Total number of deaths: 8,942,217.

## Discussion

We performed the first nationwide population-based study on Chagas mortality in Brazil considering multiple causes of death, and provided a comprehensive overview of mortality associated with Chagas' disease and trend over time. The data show that Chagas' disease is still a public health problem in the country. In general, mortality indicators tended to decline, but there were different patterns between regions. Advanced age and region of residence were identified as important risk factors for death by Chagas' disease. The presence of Chagas' disease as one of the leading causes of death among elderly in Brazil indicates that the consequences of infection acquired in the past are still present for a significant portion of this population [17].

A previous Brazilian study using deaths from Chagas' disease as the underlying cause between 1981 and 1998 found a 44.4% decrease in mortality rates due to Chagas' disease in 1981–1983 as compared to 1996–1998, and described similar regional patterns [18]. The high risk of death in Minas Gerais and Goiás states is consistent with areas of high endemicity and vector transmission in previous decades, as reflected by the percentage of infection and infestation observed in entomological and serological surveys performed in 1975–1983 [19] and 1975–1980 [20], respectively. The high mortality rates in the Federal District can be explained by intense migration of rural populations from endemic areas [18], [21]. In fact, about 1/5 of migrants in the Federal District come from rural endemic areas [21]. This scenario highlights future challenges and the need for continued disease surveillance of main transmission routes in these endemic regions [19], [22], but also for new control strategies including oral transmission in the Amazon region and secondary vectors such as *Triatoma brasiliensis* in the Northeast region [18], [19], [23]. In addition to these measures, adequate access to health services and social assistance to the many chronic chagasic patients should be guaranteed [22].

Regional differences of the number of deaths due to Chagas' disease reflect how effective the measures were adopted for the control of vector transmission and transfusion in the past, but also may be explained by other factors: transmission will need the presence of a suitable vector; population immigration of infected people will influence transmission dynamics; and there is unequal recognition of the disease, quality of care and diagnostic capacity in the different regions [24]. This is exemplified by the elimination of transmission of Chagas' disease via the main vector *T. infestans* which is not frequent in the North and Northeast and thus did not have a considerable impact on transmission dynamics in these two regions [18], [22]. This indicates that control and primary and secondary prevention of Chagas' disease in the North and Northeast has been neglected, resulting in the emergence of new cases and constant or even increasing mortality rates [25].

The fact that Chagas' disease is mostly a slowly progressive and chronic disease and that most deaths are related to an infection acquired many years earlier suggests that the number of new cases in the Northeast may have remained constant or even increased over years. Possible difficulties in controlling secondary vector species such as *T. brasiliensis* and *Triatoma pseudomaculata*, along with oral and congenital transmission may be contributing factors for the maintenance of *T. cruzi* in the Northeast region [25]. Only a small fraction of deaths were caused by acute Chagas' disease, with highest figures in the North region. Interestingly, deaths due to acute disease were increasing during the study period. In fact, in the Amazon region the majority of new cases is caused by oral transmission often leading to acute disease, mainly through the consumption of unpasteurized natural products (such as the palm products *açai* and *bacaba* juice), and an increased number of outbreaks of acute disease has been reported in recent years [26]. Our data further suggest that with the elimination of *T. infestans* as the main vector in the country, there is a need to develop sustainable control methods to reduce the chance of occurrence of cases directly dependent on secondary transmission by other vectors [9] and new strategies to control emerging oral transmission in the Amazon region [26].

Similar to previous studies, our data show that males and advanced age groups were at higher risk for death related to Chagas' disease [14], [21], [24]. However, our results did not provide sufficient evidence for the association between males and Chagas mortality. Whereas in bivariable analysis male gender appeared to be protective, in multivariable analysis being male was a risk factor for mortality. This may be caused by the fact that male sex *per se* is not a risk factor, but gender-specific behavior patterns causing a higher prevalence in males, besides social, cultural and behavioral differences between populations studied [27]. Rassi Jr. *et al*. [28] describe a controversy about the prognostic value of male mortality from Chagas' disease, shown to be associated with a worse prognosis in some studies [29], [30], but that this finding was not replicated in others [31]–[34]. However, most studies involved restricted subgroups, in contrast to our data.

The highest Chagas death rates in the older age groups corroborates previous studies [14], [21], [24] and confirms that there is a marked tendency of aging of chagasic patients. This transition can be explained mainly due to a cohort effect, a result of exposure to infection with *T. cruzi* in the past [35], considering that transmission routes of Chagas' disease (vector-borne and blood transfusion) have been widely controlled in Brazil [9], [36]. The decrease in early mortality has also been attributed to reduced reinfection rates, leading to reduced severity of clinical signs of infection [12], [14] with consequently longer survival of patients [37]. Elderly patients with Chagas' disease are a particularly vulnerable population, considering the harmful effects of a combination of Chagas' disease and other chronic degenerative diseases [17]. The frequent association of chronic disease causes significant demand for health services and medications that predispose to numerous risks, whereas the association between Chagas' disease and other chronic diseases may increase mortality and worsen the quality of life of those who are in such an unfavorable condition [17].

The risk of death due to Chagas' disease was higher among blacks and mixed race compared to the white population, indicating social disparities in the determination of death related to Chagas' disease. This fact corroborates the findings of Gonçalves *et al*. [34] that during a follow-up of a cohort of chronic chagasic patients from an endemic area found that different races or black color were poor prognostic factors for mortality due to Chagas' disease. However, in more than 10% of death certificates this information was missing, limiting the validity and reliability of this result. As there were only 54 deaths reported in indigenous people in our study, no further conclusions can be made regarding this subgroup.

Our data also point to an important topic regarding notification of Chagas' disease. Historically, only acute cases are subject to compulsory notification, reflecting the focus of control actions against Chagas' disease as an acute condition and on reduction of transmission. The Brazilian Ministry of Health has been discussing compulsory notification of chronic forms, considering the burden of chronic disease in the country and the fact that reactivation of Chagas' disease in the presence of HIV infection is considered an AIDS-defining condition in Brazil [38]. Our study will provide further evidence for the need of introduction of chronic forms as a notifiable disease.

Our study contains some limitations. The number of Chagas-related deaths may have been underreported, despite the important progress over the period under study both in the coverage of the Mortality Information System (*SIM*), and the quality of information on causes of deaths. Quality of data may also vary between regions in the country [14]. We included multiple causes of deaths, *i.e*. the mention of Chagas' disease in any field rather than only the underlying cause, to reduce this error. The sociodemographic conditions, such as race/color, education and usual occupation, considered as possible factors predictive of mortality associated with Chagas' disease, showed a considerable proportion of unknown data. Despite these limitations, we consider the results of this study of high validity and highly representative, since all death certificates during the period 1999 to 2007 were included in Brazil, a country of continental dimensions.

Our data show that mortality rates increased by 21% when multiple causes of death were considered, as compared to underlying causes of death. In fact, other authors have highlighted previously the importance of not only including underlying causes in mortality statistics, to better reflect the true epidemiology of Chagas' disease [14], [39].

We conclude that the wealth of information provided by the analysis of mortality from multiple causes contributed to the identification of the epidemiological situation of mortality associated with Chagas' disease and to predict future trends. Thus, our study provides comprehensive and reliable information for planning and evaluation of control measures of Chagas' disease in Brazil. In addition to control measures against transmission of disease, one of the main challenges is the obvious need to improve clinical care and surgery, ensuring adequate attention to the large number of cases Chagas' disease that accumulated over the last decades.
